# Molecular Diffusion-Driven Graded Polymer Hole-Transport Layers Enabling Efficient and Durable Organic Photovoltaics

**DOI:** 10.3390/polym18212636

**Published:** 2026-10-24

**Authors:** Seungpil Baek, Minwoo Nam

**Affiliations:** Department of Electronic Engineering, Keimyung University, Daegu 42601, Republic of Korea

**Keywords:** organic photovoltaics, interlayer, graded hole-transport layer, molecular diffusion, water-transfer printing, surface doping, interfacial engineering, thermal stability

## Abstract

High-performance and durable organic photovoltaics (OPVs) require interlayers that enable efficient carrier extraction and long-term stability while remaining compatible with scalable and printing-based processing. In this study, we demonstrate a molecular diffusion-driven graded polymer hole-transport layer (g-HTL) for efficient and thermally durable OPVs. The g-HTL is fabricated by water-transfer printing a thin PM6 layer onto a non-fullerene acceptor (NFA)-rich PM6:L8-BO active layer, followed by surface treatment with phosphomolybdic acid (PMA). The limited penetration of PMA produces a vertically graded p^+^–p–i configuration within the printed polymer layer, while preserving its laterally correlated surface morphology. OPVs incorporating a g-HTL achieve a power conversion efficiency of 17.18%, compared with 16.31% for conventional MoO_3_-based devices, owing to the improved carrier transport and collection. The printed PM6-based graded layer exhibits high morphological stability under thermal stress. Together with the thermally robust NFA-rich active layer, this morphologically stable g-HTL enables OPVs to retain nearly 90% of their initial efficiency after thermal aging at 85 °C for 15 days, compared with approximately 70% for MoO_3_-based devices. These results demonstrate that molecular diffusion engineering of printed polymer interlayers is an effective strategy for improving the efficiency and thermal durability of polymer-based photovoltaic devices.

## 1. Introduction

Organic photovoltaics (OPVs) have emerged as promising next-generation energy-conversion technologies because of their solution processability, mechanical flexibility, low weight, and compatibility with large-scale manufacturing [1,2,3]. Recent advances in molecular design and morphological control have enabled the development of high-performance OPVs based predominantly on bulk heterojunction (BHJ) active layers composed of polymer donors and non-fullerene acceptors (NFAs) [3,4]. A typical OPV architecture integrates the polymer: NFA BHJ between two electrodes, together with charge-selective interlayers that facilitate the transport and extraction of photogenerated carriers. Among these interlayers, the layer deposited directly onto the upper surface of the active layer, typically the hole-transport layer (HTL) in an inverted device, is particularly important because its formation affects both the electrical characteristics and interfacial structure of the underlying BHJ active layer [5,6,7]. Moreover, in advanced NFA systems based on Y6 and its derivatives, intra-moiety excited states associated with intermolecular electronic coupling and excited-state delocalization can facilitate charge generation even at relatively small energetic offsets [8,9]. Under such conditions, the efficient transport and collection of photogenerated carriers before recombination become increasingly important for photovoltaic performance, further emphasizing the role of the interlayer in establishing efficient and stable charge-extraction pathways [10].

An effective HTL must satisfy stringent requirements to enable high-performance OPVs. It should possess suitable interfacial electronic properties for selective hole extraction while suppressing electron leakage, charge accumulation, and recombination loss [10,11]. At the same time, the HTL should form continuous and uniform contact with adjacent layers because pinholes, locally disconnected regions, or pronounced spatial variations in surface properties produce nonuniform electrode contact and localized charge-extraction losses [12]. From a manufacturing perspective, the HTL should be compatible with scalable coating or printing processes [11]. Equally important, the deposition and subsequent modification should minimally disturb the molecular organization and nanoscale morphology of the underlying BHJ [6,13]. Beyond the initial device performance, both the HTL and its interfaces should remain structurally and electronically stable over time, so that the charge transport and extraction characteristics established in the fresh device are preserved during prolonged operation [14,15]. Simultaneously satisfying these electrical, morphological, processing, and stability requirements remains a major challenge in the development of efficient, durable, and scalable OPVs.

Thermally evaporated MoO_3_ has been widely employed as the HTL in inverted OPVs owing to its high work function and effective hole selectivity [16]. However, the MoO_3_/BHJ interface is particularly susceptible to thermally induced degradation [13,14,17]. During thermal evaporation, MoO_3_ species can penetrate into or interact with the upper region of the organic active layer, altering the local interfacial properties. Their distribution can evolve further during prolonged thermal exposure, potentially inducing additional chemical and electronic interactions with the donor and acceptor components [7,13,14,18]. In our previous studies, thermally induced increases in charge trapping and recombination were associated with the penetration and redistribution of MoO_3_ species near the upper active layer interface, indicating that interfacial deterioration is a major contributor to thermal burn-in degradation in OPVs [19,20]. Consistent with this interpretation, we demonstrated that replacing MoO_3_ with a surface-doped polymer HTL reduced the trap density and substantially improved the thermal stability of the resulting OPVs [20]. Beyond these intrinsic interfacial stability issues, vacuum-based MoO_3_ deposition increases fabrication complexity and production costs and is less compatible with commercial manufacturing based on scalable coating and printing processes [5,21]. Direct deposition onto solution-processed BHJ films may also modify the near-surface region of the active layer and create an extended interfacial region, potentially compromising device reproducibility and long-term interfacial stability [6,22]. These limitations have motivated the development of solution-processable organic and inorganic HTLs [21,23]. However, many alternative HTLs exhibit insufficient conductivity, limited processing tolerance, poor interfacial contact, and inadequate thermal stability. Importantly, most conventional interlayer designs retain a discrete boundary between the BHJ and HTL, where abrupt compositional and electrical variations promote charge accumulation and hinder effective carrier extraction [10,24]. These limitations underscore the need for alternative HTL concepts that enable controlled interfacial structures and electronic properties while remaining compatible with scalable fabrication.

In this study, we demonstrate a molecular diffusion–driven graded polymer HTL (g-HTL) that enables efficient and thermally durable hole extraction in OPVs. A thin PM6 film was deposited onto an NFA-rich PM6:L8-BO BHJ via water-transfer printing, followed by post-deposition treatment with phosphomolybdic acid (PMA). Surface doping increased the hole density near the upper region of the printed PM6 layer, whereas dopant diffusion over a limited depth resulted in a gradual decrease in the doping level through the printed PM6 layer, establishing a vertically graded p^+^–p–i configuration toward the underlying active layer. This graded electronic structure established an efficient hole transport and extraction pathway, resulting in improved photovoltaic performance compared with that of conventional MoO_3_-based reference devices. Importantly, both the NFA-rich active layer and printed PM6-based graded layer exhibited only minor morphological evolution during prolonged thermal aging. Preserving this morphology maintained the favorable charge transport and extraction characteristics of pristine devices under thermal stress, suppressing burn-in degradation and significantly enhancing the long-term stability relative to the MoO_3_-based reference. This vacuum-free and printing-compatible strategy provides a promising platform for simultaneously achieving efficient charge extraction, thermal durability, and scalable fabrication of OPVs.

## 2. Materials and Methods

### 2.1. Fabrication of the g-HTL

The g-HTL was prepared by combining the water-transfer printing of PM6 with subsequent surface doping using PMA. For water-transfer printing, 15 μL of a PM6 solution (4 mg mL–1 in chlorobenzene) was dispensed onto the surface of deionized water. The solution spontaneously spread over the water surface and formed a floating PM6 film after the solvent evaporated. The NFA-rich PM6:L8-BO layer was brought in contact with the floating film, allowing the PM6 layer to be transferred onto the active layer. The resulting printed PM6 overlayer is approximately 25 nm thick. The stacked PM6:L8-BO/PM6 films were thermally annealed at 100 °C for 10 min. Surface p-type doping of the transferred PM6 layer was performed at room temperature, following a previously reported procedure [20]. A 0.5 M PMA solution was prepared in an isopropyl alcohol:1,2–dichlorobenzene mixed solvent (99:1 *v*/*v*) and spin-coated onto the printed PM6 layer. The PMA-treated films were then rinsed with pure isopropyl alcohol for 1 min. The limited penetration of PMA into the film surface generated a depth-dependent p-doped region within the upper portion of the printed PM6 layer, forming a g-HTL.

### 2.2. OPV Fabrication

The indium tin oxide (ITO)-coated glass substrates were sequentially cleaned by ultrasonication in deionized water, acetone, and isopropyl alcohol for 10 min each, followed by ultraviolet (UV)–ozone treatment for 15 min. The ZnO precursor solution was then spin-coated onto the cleaned ITO surface and thermally annealed at 300 °C for 10 min to form an electron transport layer. The active layer was deposited onto the ZnO-coated substrates by spin-coating a chloroform solution containing PM6 and L8-BO at a donor-to-acceptor weight ratio of 3:7 [25]. The g-HTL was subsequently formed on the active layer using water-transfer printing and PMA surface-doping procedures, as described above. After formation of the g-HTL, a 100 nm Ag electrode was deposited by thermal evaporation under high vacuum (<10^−6^ Torr). The resulting device architecture was ITO/ZnO/PM6:L8-BO/g-HTL/Ag. For comparison, reference devices were fabricated using the same process, except that the g-HTL was replaced with a thermally evaporated MoO_3_ HTL. The corresponding reference device was composed of ITO/ZnO/PM6:L8-BO/MoO_3_/Ag. PM6 and L8-BO (Ossila, Sheffield, UK) and PMA (Alfa Aesar, Ward Hill, MA, USA) were used as received without further purification. All solvents were purchased from Sigma-Aldrich (St. Louis, MO, USA) and used as received.

### 2.3. Characterization

Ultraviolet–visible (UV–Vis) spectra were collected using an OPTIZEN POP spectrophotometer (KLAB, Daejeon, Republic of Korea). X-ray photoelectron spectroscopy (XPS) was performed using a Nexsa Surface Analysis System (Thermo Fisher Scientific, Waltham, MA, USA), and depth-dependent chemical information was obtained by sequential ion-etching measurements. The surface topography and phase characteristics were examined using a non-contact-mode atomic force microscopy (AFM, XE-150, Park Systems, Gwacheon, Republic of Korea). All AFM scans were acquired over an area of 2 μm × 2 μm. For each condition, two distinct areas were measured on each of two films, yielding four area measurements. Height and phase *R*_q_ were evaluated and reported as the mean ± standard deviation across these four measurements. Representative images were selected based on the *R*_q_ values closest to the corresponding mean for each condition. The AFM topography data were further evaluated using the radially averaged autocorrelation functions (ACFs) and an ABC power spectral density (PSD) model [26,27]. The PSD profiles represent averages of four AFM measurements per condition. Photovoltaic measurements of the OPV devices were performed using a Keithley 2450 source-measure unit (Tektronix, Beaverton, OR, USA) under simulated air mass 1.5 global (AM 1.5G) irradiation at 100 mW cm^−2^, generated by a SimuLight SS-LED50S solar simulator (McScience, Suwon, Republic of Korea). Incident light intensity (*P*_light_) was calibrated using a certified Si reference cell (S301-K125; McScience, Suwon, Republic of Korea). For *P*_light_-dependent measurements, reflective neutral-density filters (Thorlabs, Newton, NJ, USA) were used to vary *P*_light_. The hole-transport properties were investigated using hole-only devices with ITO/poly(3,4-ethylenedioxythiophene):poly(styrene sulfonate) (PEDOT:PSS)/PM6:L8-BO/MoO_3_/Ag and ITO/PEDOT:PSS/PM6:L8-BO/g-HTL/Ag. The effective hole mobility was extracted from the space-charge-limited current (SCLC) region using the Mott–Gurney relation, whereas trap-state density (*N*_t_) was determined from the trap-filled-limit voltage (*V*_TFL_) [28,29,30]. For the thermal stability measurements, the devices were encapsulated using UV-curable epoxy and aged on a digital hot plate at 85 °C in ambient air in the dark. At selected time points, the devices were removed from the hot plate and allowed to cool to room temperature before *J*–*V* measurements under AM 1.5G illumination at 100 mW cm^−2^. After each measurement, the devices were returned to the hot plate to resume aging at 85 °C.

## 3. Results and Discussion

**Figure 1:**
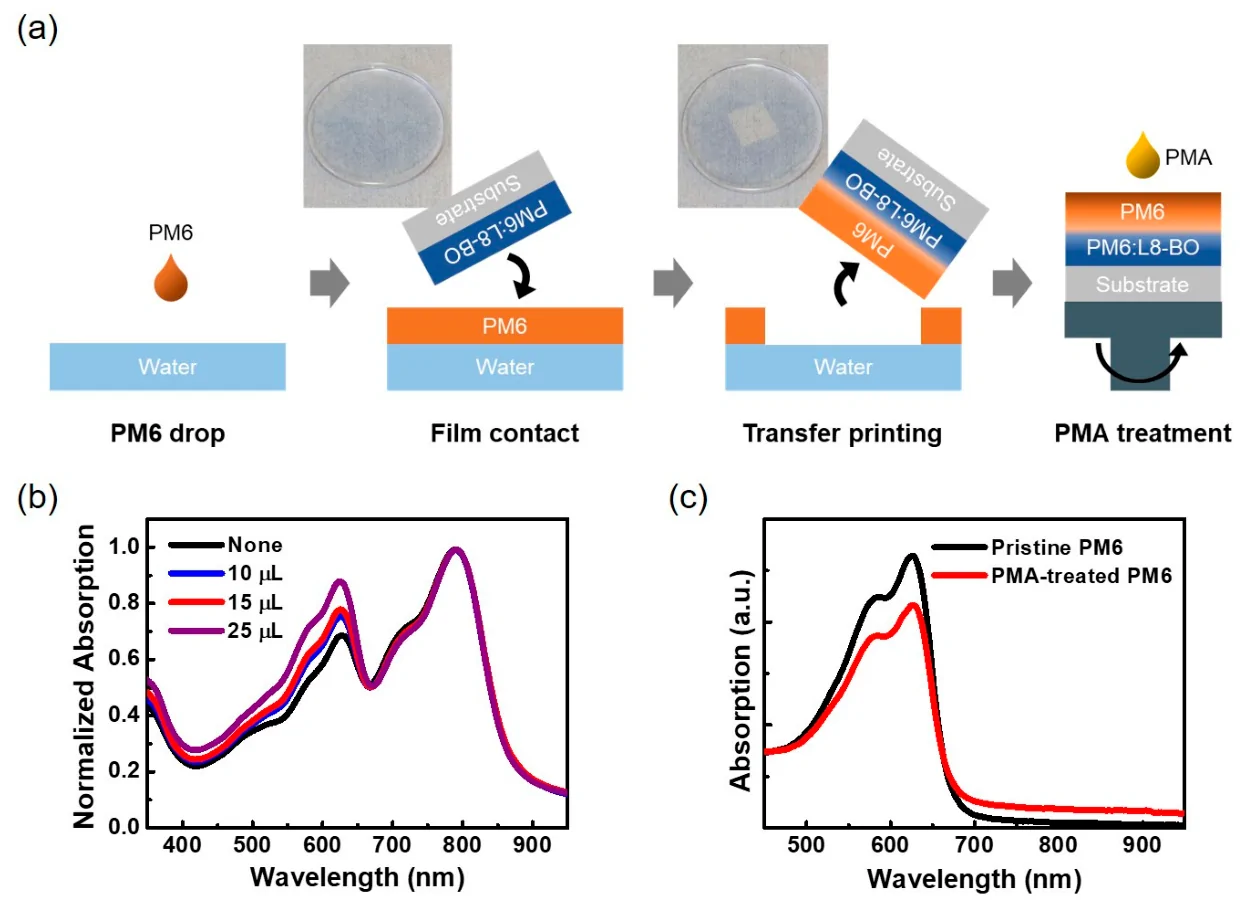
(**a**) Schematic of the fabrication process of the graded polymeric layer, including photographs of the water-transfer printing process. (**b**) Normalized UV–Vis absorption spectra of PM6:L8-BO/PM6 stacked films prepared using various volumes of PM6 solution during water-transfer printing. (**c**) UV–Vis absorption spectra of the printed PM6 film before and after PMA treatment, showing the spectral features associated with surface-confined oxidative p-type doping.

Figure 1a illustrates the fabrication concept of a diffusion-driven graded HTL. An NFA-rich PM6:L8-BO BHJ film with a donor-to-acceptor weight ratio of 3:7 was selected as the active layer because this composition exhibited favorable photovoltaic performance and thermal stability [25]. A thin PM6 overlayer was deposited onto the NFA-rich PM6:L8-BO blend by using a transfer printing method on water, followed by thermal annealing at 100 °C for 10 min [20,31]. The photographs in Figure 1a show the successful transfer of the floating PM6 film onto the underlying substrate, demonstrating the applicability of the water-transfer printing process for the formation of the polymer overlayer. The exposed surface of the printed PM6 layer was subsequently p-doped via a post-deposition treatment with PMA. Because PMA penetrates only to a limited depth from the film surface, the doping level is expected to be highest near the upper surface and decrease with increasing depth in the PM6 layer [32,33]. Spatially limited surface doping is, therefore, expected to produce a gradual compositional and electronic transition, giving rise to a vertically graded p^+^–p–i configuration from the upper surface toward the active layer. This graded configuration was intended to establish a continuous pathway for hole transport and extraction toward the Ag electrode without using a thermally evaporated MoO_3_ layer. Further details of the fabrication procedure are provided in Section 2.

Figure 1b shows the normalized UV–Vis absorption spectra of the PM6:L8-BO/PM6 stacked films prepared using various volumes of PM6 solution during the water-transfer printing process. Each spectrum was normalized to the principal absorption peak of L8-BO. Following incorporation of the PM6 overlayer, the absorption shoulder near 630 nm became more pronounced. This spectral feature is associated with the characteristic absorption of PM6 and confirms successful stacking of the PM6 film onto the active layer. As the dispensed volume of the PM6 solution increases, the relative absorption intensity near 630 nm gradually increases, indicating an increase in the amount of PM6 deposited on the underlying BHJ. These results demonstrate that the printed PM6 overlayer can be readily controlled by varying the volume of the solution used to form a floating film on water. A dispensed volume of 15 μL was used for the subsequent experiments, yielding a printed PM6 overlayer with a thickness of approximately 25 nm. This condition was selected to form a continuous polymer overlayer while avoiding an excessively thick transport region, which could introduce additional resistance to hole extraction [34].

Figure 1c shows a comparison of the absorption spectra of the printed PM6 layer before and after surface treatment with PMA. Following PMA treatment, the characteristic visible absorption of neutral PM6 decreased, accompanied by the emergence of additional absorption in the near-infrared (NIR) region. The reduction in visible absorption is attributed to the bleaching of the neutral PM6 absorption bands, whereas the NIR absorption originates from polaronic states generated by the oxidative p-doping of PM6 [32,33,35]. The work function of the printed PM6 film increased from approximately 4.91 to 5.15 eV after PMA treatment (Figure S1). This increase is consistent with a deeper Fermi level relative to the vacuum level and, together with the NIR polaronic absorption, supports oxidative p-type doping of PM6 [19,20,32]. The simultaneous presence of neutral polymer and polaronic absorption features is consistent with the spatially nonuniform doping induced by the limited penetration of PMA into the printed PM6 layer. Combined with the interfacial transition formed between the BHJ and the pristine PM6 layer, this surface-confined doping provides the basis for the proposed vertically graded electronic structure of the HTL, which is examined in greater detail by depth-resolved XPS analysis in the following section.

**Figure 2:**
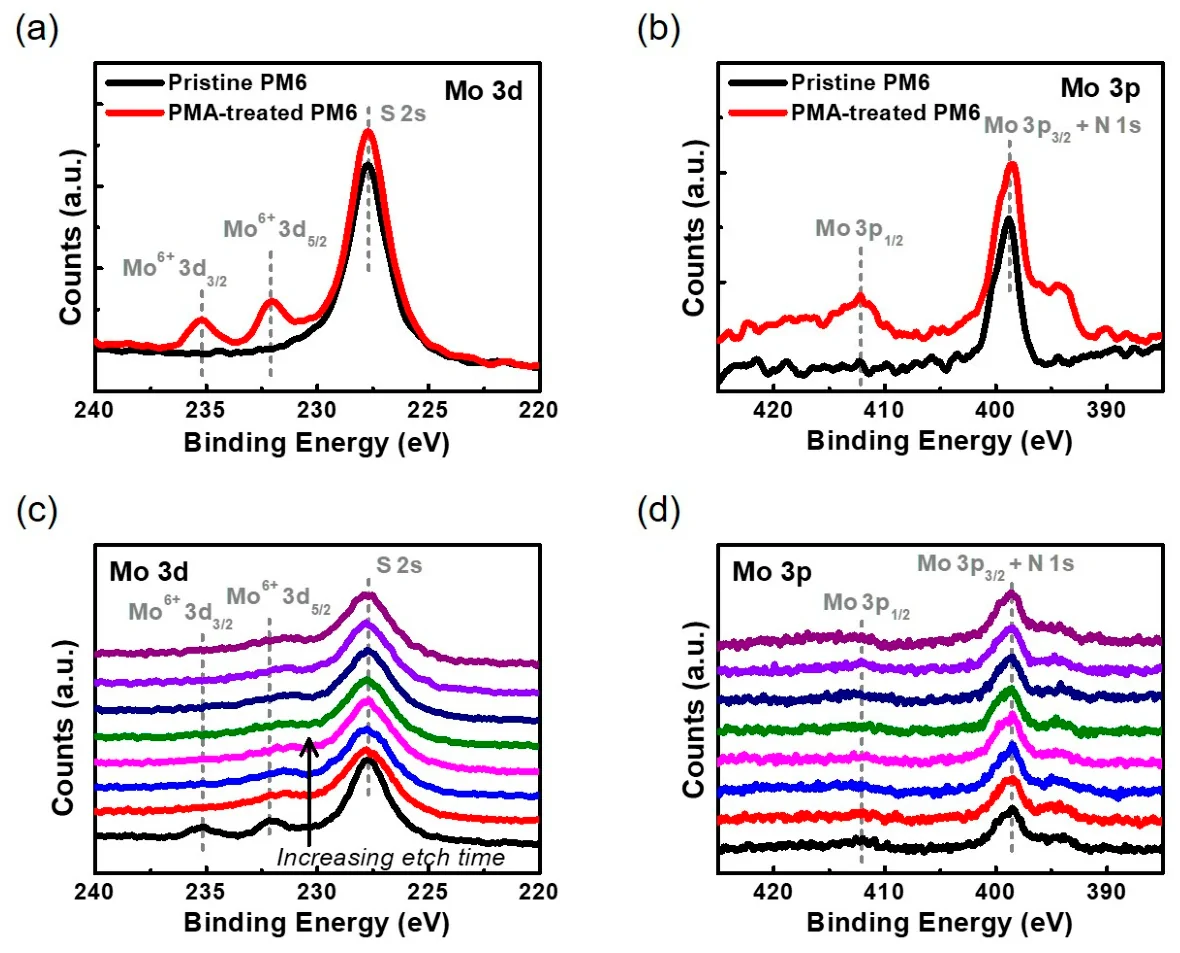
XPS characterization of the PMA-treated printed PM6 layer. (**a**) Surface Mo 3d and (**b**) Mo 3p/N 1s spectra of pristine and PMA-treated printed PM6 films. (**c**) Depth-resolved Mo 3d and (**d**) Mo 3p/N 1s spectra of the PMA-treated printed PM6 film at selected etching times. The spectra are vertically offset for clarity, with increasing etching time corresponding to increasing analysis depth.

To verify the formation of the graded p-type PM6 layer, XPS measurements were performed on PMA-treated and untreated printed PM6 films. Figure 2a and Figure 2b show the surface Mo 3d and Mo 3p spectra of the films, respectively. In the Mo 3d region, the pristine PM6 film exhibited no discernible Mo-related features, whereas the PMA-treated PM6 film exhibited distinct peaks at approximately 232 and 235 eV, which were assigned to the Mo^6+^ 3d_5/2_ and Mo^6+^ 3d_3/2_ components, respectively [36]. The pronounced Mo^6+^ doublet provides direct evidence of the successful incorporation of PMA-derived species into the printed PM6 layer. Consistently, an additional Mo 3p_1/2_ feature was observed at approximately 412 eV for the PMA-treated film, whereas the spectral region near 399 eV contained contributions from the N 1s and adjacent Mo 3p_3/2_ components [37]. The appearance of Mo-related features in the two independent core-level regions provided complementary evidence of the presence of PMA-derived Mo species in the printed PM6 film.

To further examine the vertical distribution of the PMA-derived species, Figure 2c and Figure 2d show the depth-resolved Mo 3d and Mo 3p spectra of the PMA-treated PM6 film at selected etching times, respectively. The spectra were vertically offset for clarity, with increasing etching time shown from bottom to top. The corresponding depth-resolved spectra of the pristine PM6 film showed no discernible Mo-related features over the same etching range (Figure S2), confirming that the Mo-related spectral evolution observed after PMA treatment originated from the incorporated PMA-derived species rather than from the underlying organic semiconductor layers. Pronounced depth-dependent evolution was observed in the PMA-treated films. At the outermost surface, the Mo^6+^ 3d_5/2_ and 3d_3/2_ components are resolved, indicating a strong contribution from the well-defined PMA-derived Mo(VI) species near the film surface. As the etching depth increased, the characteristic Mo 3d doublet became broader and less distinctly resolved, implying that the relative contribution and local chemical environment of the PMA-derived Mo species varied along the vertical direction rather than remaining uniform throughout the printed PM6 layer. A corresponding depth-dependent evolution was observed in the Mo 3p region, where the characteristic Mo-related features become less distinct as the etching depth increases. The mutually consistent changes observed in the two independent Mo core-level regions demonstrate the vertically nonuniform distribution and chemical environment of the PMA-derived species within the printed PM6 layer.

Importantly, the detection of Mo-related signals beneath the outermost surface indicates that the PMA treatment does not simply form a discrete layer confined to the film surface. Instead, the PMA-derived species penetrated the printed PM6 layer over a finite depth, exhibiting a pronounced depth-dependent spectral characteristic. This behavior is consistent with previous reports showing that solution-processed PMA can diffuse into conjugated polymer films over a limited depth and form p-doped regions rather than remaining exclusively on the surface [32,33,38]. In the present film, the well-resolved PMA-associated Mo(VI) surface features and their depth-dependent spectral evolution indicate that PMA-induced modification is most pronounced near the surface and attenuates toward the interior of the printed PM6 layer. This depth-dependent doping provided a gradual transition in the electronic characteristics of PM6 from the strongly modified surface region to the underlying active layer, establishing the intended graded HTL.

**Figure 3:**
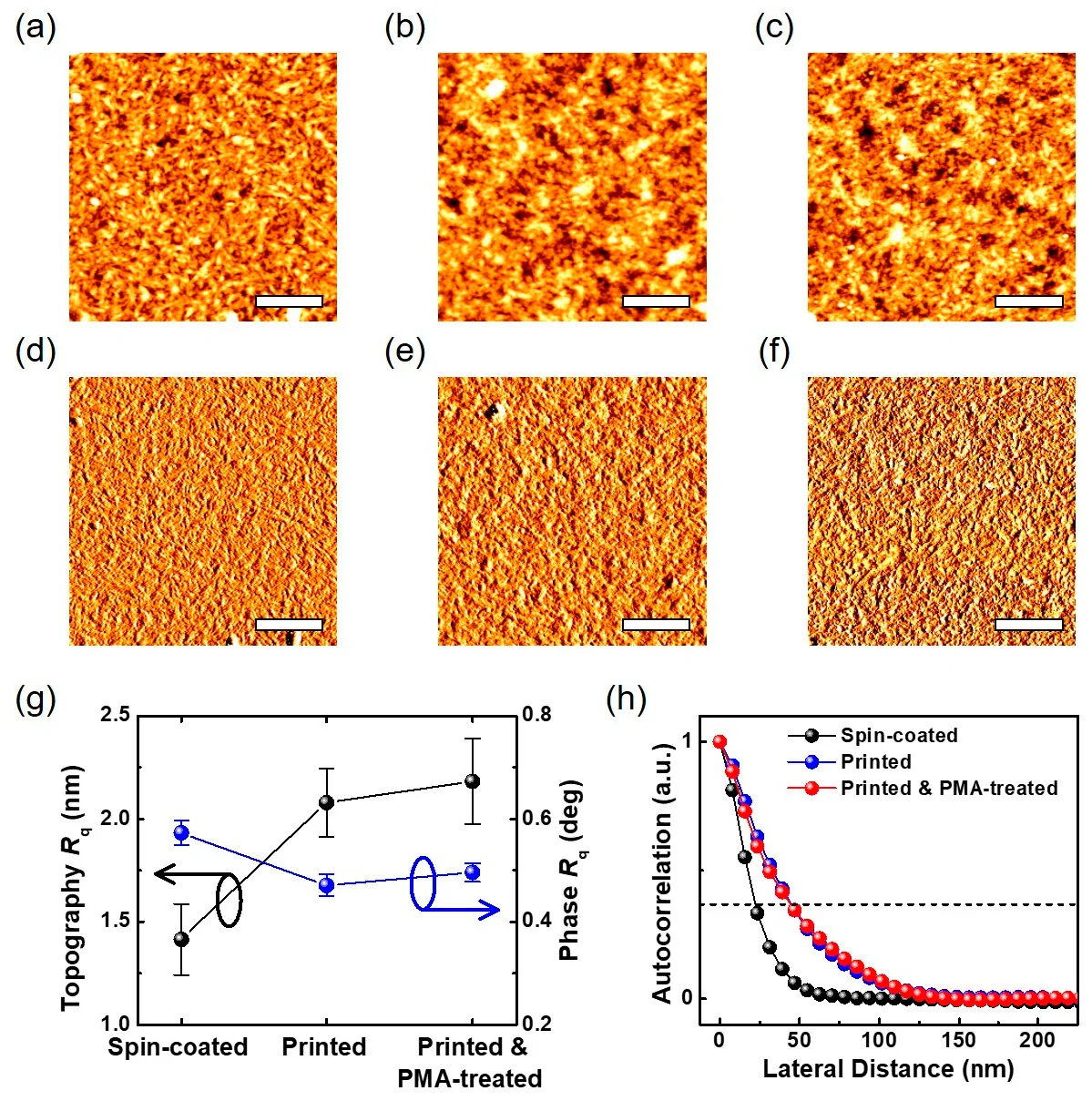
AFM topography and phase images of (**a**,**d**) spin-coated PM6, (**b**,**e**) water-transfer-printed PM6, and (**c**,**f**) water-transfer-printed PM6 subsequently treated with PMA. The scan size is 2 μm × 2 μm, and scale bars represent 500 nm. (**g**) Mean topographic and phase *R*_q_ values with error bars indicating standard deviations across four AFM images per condition. (**h**) Normalized radially averaged ACF curves extracted from the AFM topography images. The horizontal dashed line indicates the 1/e autocorrelation level used to determine the lateral correlation length (*ξ*).

The preceding XPS analysis confirms the intended p^+^–p–i configuration of the vertically graded PM6 layer formed through the combined transfer-printing and PMA surface-doping processes. In addition to an appropriate depth-dependent electronic structure, the effective operation of an organic semiconducting film as a charge transport layer requires a continuous and sufficiently uniform morphology that supports lateral and vertical charge migration [39,40]. By contrast, morphological defects such as pinholes, excessive height variations, or strong spatial variations in the surface properties produce nonuniform interfacial contact, promote charge trapping, and hinder efficient hole extraction across the device [12]. The importance of these morphological characteristics led us to examine the surface morphology of the PM6 interlayers. Figure 3a–f show the AFM topography and phase images of spin-coated PM6, water-transfer-printed PM6, and printed PM6 subsequently surface doped with PMA. The spin-coated PM6 film exhibits a relatively smooth surface with small height variations, whereas the printed PM6 film exhibits more pronounced nanoscale topographic features. This difference was quantitatively evaluated using root-mean-square roughness (*R*_q_), as summarized in Figure 3g. Height and phase *R*_q_ were evaluated separately and reported as mean ± standard deviation across four area measurements per condition. The topographic *R*_q_ increased from 1.41 (± 0.17) nm for the spin-coated PM6 film to 2.08 (± 0.16) nm for the printed PM6 film, indicating that transfer printing produced a moderately rough polymer surface. After PMA treatment, the topographic *R*_q_ remained at a comparable level, increasing only slightly to 2.18 (± 0.20) nm. These results indicate that surface doping with PMA caused only minor changes in the height profile of the printed PM6 film.

Interestingly, despite the increase in the topographic *R*_q_ after transfer printing, the phase *R*_q_ values remained comparable among the three samples (Figure 3g). This suggests that increased surface roughness was not accompanied by pronounced spatial variations in the local AFM phase response. In particular, the phase images show no sharply separated domains, isolated regions with markedly different phase contrasts, or obvious discontinuities in the film coverage. Thus, although transfer printing changed the surface topography, the printed PM6 layer retained a uniform distribution of surface properties over the lateral length scale, as probed using AFM. To further evaluate the lateral organization of the surface features, radially averaged ACF curves were extracted from the AFM topography images (Figure 3h) [41,42]. The horizontal dashed line indicates the 1/e autocorrelation level used to determine the lateral correlation length (*ξ*). Here, *ξ* was defined as the first lateral distance at which the ACF decreased to 1/e and was extracted using linear interpolation between adjacent data points. The spin-coated PM6 film exhibited a relatively rapid ACF decay, indicating that its surface height features lost their spatial correlation over a comparatively short lateral distance, with *ξ* = 22.2 nm. By contrast, both the pristine and PMA-doped printed PM6 films exhibited similar ACF profiles with slower decay, indicating a longer-range lateral correlation of their surface features, with *ξ* values of 44.7 and 44.4 nm, respectively. Thus, transfer printing approximately doubled the lateral correlation length, while PMA treatment produced little changes in this characteristic length. Moreover, the comparable *R*_q_ and *ξ* values before and after PMA treatment support the preservation of the characteristic surface roughness and lateral correlations during doping. Collectively, the PMA-doped printed PM6 layer combined a vertically graded p-type electronic structure with surface height features showing a longer lateral correlation length than those of the spin-coated PM6 film, providing a favorable interfacial structure for subsequent hole transport and extraction.

**Figure 4:**
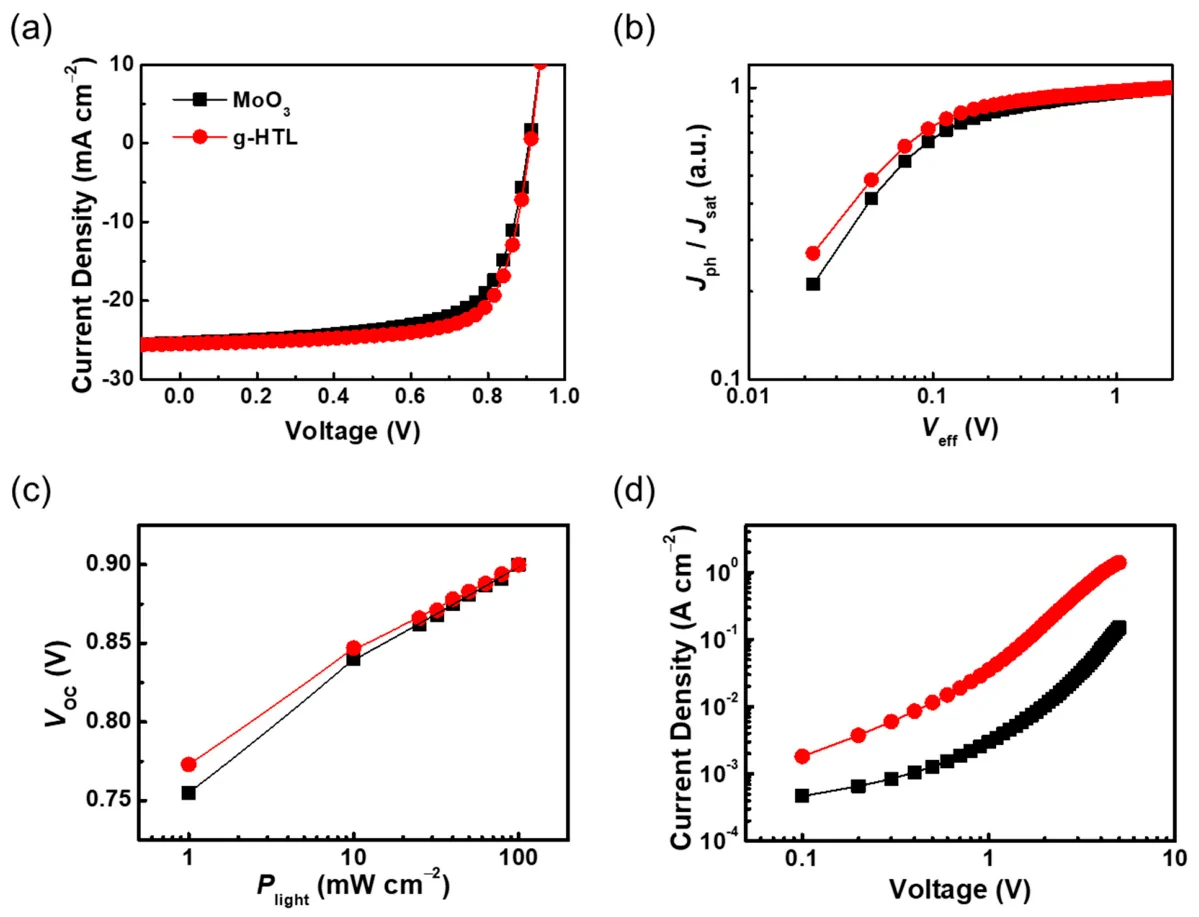
Photovoltaic and charge transport characteristics of OPVs incorporating the g-HTL compared with conventional MoO_3_-based devices. (**a**) *J*–*V* characteristics under AM 1.5G illumination at 100 mW cm^−2^. (**b**) Normalized *J*_ph_/*J*_sat_ ratio as a function of *V*_eff_. (**c**) *V*_OC_ as a function of *P*_light_. (**d**) Dark *J*–*V* characteristics of the corresponding hole-only devices analyzed using the SCLC model.

The photovoltaic characteristics of the OPVs incorporating the diffusion-driven graded polymer HTL, namely g-HTL, were compared with those of the reference devices. The g-HTL OPV was comprised of ITO/ZnO/PM6:L8-BO (3:7)/g-HTL/Ag, whereas the MoO_3_ HTL OPV consisted of ITO/ZnO/PM6:L8-BO (3:7)/MoO_3_/Ag. Figure 4a shows the current density–voltage (*J*–*V*) characteristics measured under AM 1.5G illumination at 100 mW cm^−2^. The MoO_3_-based reference device exhibited an open-circuit voltage (*V*_OC_) of 0.90 V, short-circuit current density (*J*_SC_) of 25.45 mA cm^−2^, fill factor (FF) of 71.14%, and power conversion efficiency (PCE) of 16.31%. Replacing MoO_3_ with g-HTL increased the PCE to 17.18%, mainly owing to an increase in *J*_SC_ to 25.60 mA cm^−2^ and FF to 74.62%, whereas the *V*_OC_ remained nearly unchanged at 0.90 V. The improvements in *J*_SC_ and FF indicate more efficient carrier collection and reduced transport-related losses on the hole-collecting side of the device. Statistical analysis of 18 independently fabricated devices further showed that the g-HTL OPVs exhibited narrow distributions of *V*_OC_, *J*_SC_, FF, and PCE (Figure S3), demonstrating the reproducibility of the solution-processed g-HTL interface.

To determine the origin of the enhanced *J*_SC_ and FF, the charge separation, recombination, and collection characteristics were further examined. Figure 4b shows the ratio of the photocurrent density to saturation photocurrent density (*J*_ph_/*J*_sat_) as a function of the effective voltage (*V*_eff_). Here, *J*_ph_ is defined as *J*_L_ − *J*_D_, where *J*_L_ and *J*_D_ are the current densities under illumination and in the dark, respectively; *J*_sat_ is the saturation photocurrent density obtained at a sufficiently high *V*_eff_; and *V*_eff_ is defined as *V*_0_ − *V*_a_, where *V*_0_ is the voltage at which *J*_ph_ = 0 and *V*_a_ is the applied voltage [28]. The *J*_ph_/*J*_sat_ ratio represents the fraction of photogenerated carriers contributing to the collected photocurrent under a given internal electric field [43]. The g-HTL device exhibited higher *J*_ph_/*J*_sat_ values than the MoO_3_-based reference in the low-*V*_eff_ region, where the carrier collection became increasingly sensitive to transport, recombination, and extraction losses [28,43]. At *V*_eff_ values of 0.10 and 0.05 V, the *J*_ph_/*J*_sat_ ratios of the g-HTL device were 0.77 and 0.57, respectively, compared with 0.66 and 0.46 for the MoO_3_-based device. By contrast, the two devices exhibited comparable *J*_ph_/*J*_sat_ values near short-circuit conditions, suggesting similar overall charge generation and separation efficiencies under a sufficiently strong internal field. The greater difference at low *V*_eff_, therefore, indicates that the main advantage of the g-HTL emerges under weaker-field operating conditions, where efficient carrier transport and interfacial extraction become increasingly important. This behavior is consistent with the higher FF of the g-HTL OPV. Figure 4c shows the dependence of *V*_OC_ on the *P*_light_, which was analyzed to assess the relative contributions of different recombination pathways [44]. In the *P*_light_ range of 10–100 mWcm^−2^, the g-HTL OPV exhibited a slope of 1.02 *kT*/*q*, lower than the value of 1.15 *kT*/*q* obtained for the MoO_3_-based reference. The difference became more pronounced in the lower-intensity range of 1–10 mWcm^−2^, where slopes of 1.53 and 1.88 *kT*/*q* were obtained for the g-HTL and MoO_3_-based OPVs, respectively. A *V*_OC_-ln(*P*_light_) slope close to *kT*/*q* is generally associated with predominantly bimolecular recombination between free carriers, whereas a larger slope approaching 2 *kT*/*q* indicates an increasing contribution from trap-assisted recombination [44,45]. Accordingly, the consistently smaller slopes observed for the g-HTL device, particularly at low light intensities where trap-mediated processes become relatively more influential, indicate a reduced contribution from trap-assisted recombination.

To further examine hole transport at the anode side, dark *J*–*V* characteristics were measured using hole-only devices with the structures ITO/PEDOT:PSS/NFA-rich BHJ/g-HTL or MoO_3_/Ag (Figure 4d). Based on the Mott–Gurney SCLC model, the device incorporating the g-HTL exhibited an effective hole mobility of 6.7 × 10^−4^ cm^2^ V^−1^s^−1^, substantially higher than the 1.5 × 10^−4^ cm^2^ V^−1^s^−1^ obtained for the MoO_3_-based reference [28,29]. Because the extracted mobility represents the effective hole transport through the active layer and hole-collecting contact as an integrated device stack, a higher value indicates reduced resistance to hole transport and extraction in the g-HTL device [46]. The *N*_t_ was estimated from the *V*_TFL_ according to $V_{\mathrm{TFL}}=\frac{eN_{t}L^{2}}{2\varepsilon \varepsilon_{0}}$, where *e* is the elementary charge, *L* is the thickness of the active layer, *ε* is the relative dielectric constant, and ε_0_ is the vacuum permittivity [30]. The g-HTL device exhibited an *N*_t_ of 2.8 × 10^16^ cm^−3^, which is considerably lower than the 3.8 × 10^16^ cm^−3^ obtained for the MoO_3_-based device. This reduction indicates a lower density of electrically active traps in the device stack incorporating the graded interface, consistent with the reduced contribution of trap-assisted recombination inferred from the *V*_OC_−ln(*P*_light_) analysis and the improved carrier-collection behavior at low *V*_eff_. Taken together, these results indicate that g-HTL does not substantially alter bulk charge generation, while improving effective hole transport and carrier collection and reducing trap-mediated recombination losses at the hole-collecting side of the device. These electrical advantages account for the simultaneous increases in *J*_SC_ and FF, and consequently, the higher PCE of the g-HTL OPVs.

**Figure 5:**
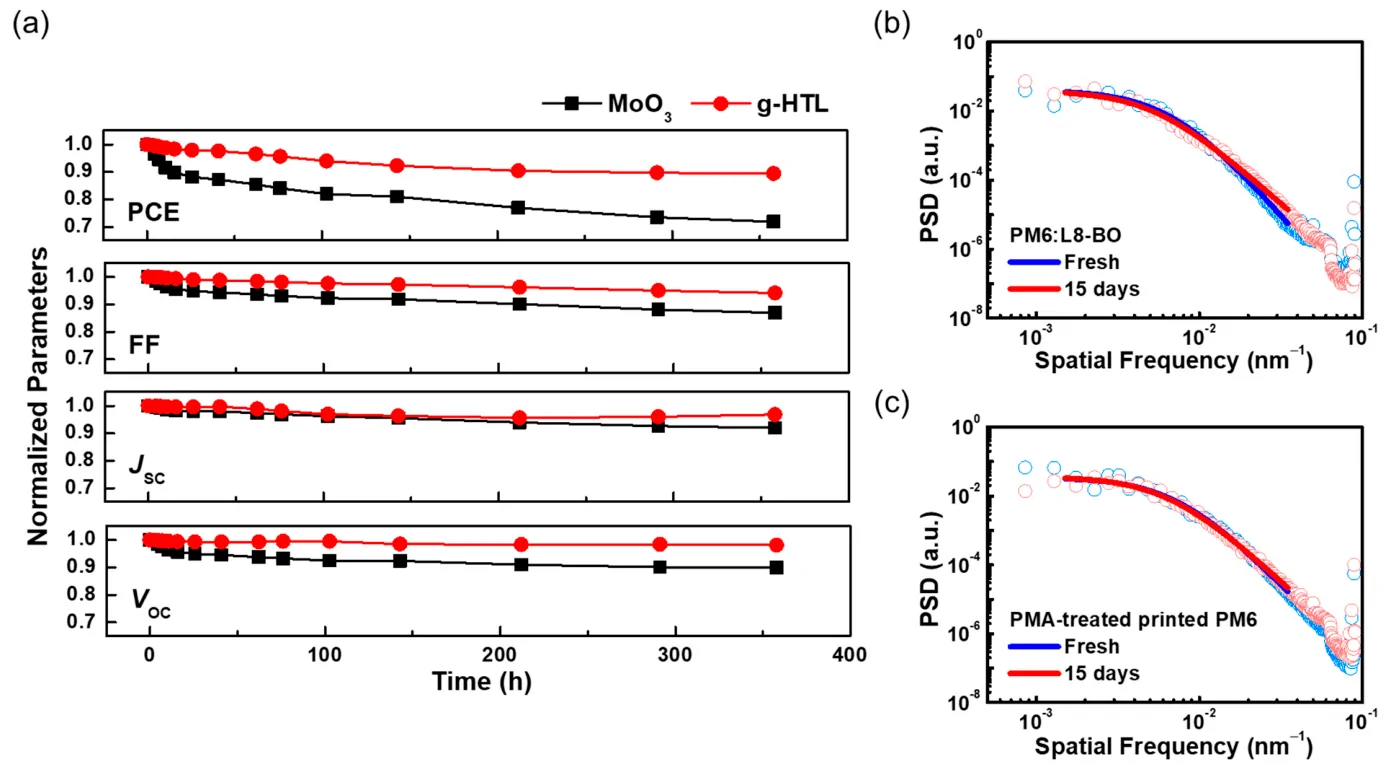
(**a**) Time-dependent evolution of the normalized photovoltaic parameters of g-HTL and MoO_3_-based OPVs during thermal aging at 85 °C. The plotted data represent mean values from six devices per architecture. PSD analysis of the AFM topography data using the ABC model for (**b**) the NFA-rich PM6:L8-BO active layer and (**c**) the g-HTL before and after thermal aging at 85 °C for 15 days. Open symbols in (**b**,**c**) represent mean PSD profiles from four measurements per condition, and solid lines represent the ABC fits.

The preceding analyses demonstrate that the diffusion-driven g-HTL can replace the conventional MoO_3_ HTL while providing improved photovoltaic performance. To assess the viability of this interfacial design further, its thermal stability was evaluated under accelerated aging conditions. Thermal stress was selected because it induces structural rearrangement within organic semiconductor films and accelerates degradation at charge-selective interfaces, both of which directly affect charge transport, recombination, and extraction [47,48]. Figure 5a shows the time-dependent evolution of the photovoltaic parameters during thermal aging at 85 °C. The plotted data represent mean values from six devices per architecture, with each parameter normalized to its corresponding initial value. The g-HTL OPVs exhibited substantially greater thermal durability than their MoO_3_-based counterparts, with both initial burn-in loss and subsequent long-term degradation being suppressed. After 15 days of aging, the g-HTL devices retained nearly 90% of their initial PCEs, whereas the MoO_3_-based reference devices retained only approximately 70% of their initial PCEs. For a quantitative lifetime comparison, T_80_ was defined as the time at which the PCE decreased to 80% of its initial value. Linear interpolation across the 80% threshold in the normalized PCE curve yielded an estimated T_80_ of 143 h for the MoO_3_-based devices. The g-HTL devices remained above this threshold throughout the observation period (T_80_ > 360 h). The improved PCE retention of the g-HTL OPVs was primarily associated with a more stable *V*_OC_ and FF, suggesting that the charge transport and extraction characteristics at the hole-collecting side were more effectively preserved under thermal stress [13,14,15].

The thermally induced morphological evolution of organic semiconducting layers was examined to identify the origin of the prolonged device lifetime. Figure S4 shows the AFM images of the NFA-rich PM6:L8-BO active layer and g-HTL before and after thermal aging at 85 °C for 15 days. The corresponding topographic and phase *R*_q_ values are summarized in Figure S5. Only small changes in both parameters were observed after aging, indicating that prolonged heating caused little variation in the surface height distribution or local phase contrast of either film. For a more quantitative evaluation of various lateral length scales, the AFM topography data were further analyzed using the ABC PSD model, as shown in Figure 5b,c. This model describes the surface morphology in terms of the magnitude of height fluctuations, lateral correlation of surface features, and contribution of roughness at finer lateral scales across a broad spatial frequency range [26,27]. Lower and higher spatial frequencies correspond to larger and finer surface features, respectively. In the ABC model, A represents the PSD amplitude at low frequencies, B sets the characteristic lateral scale associated with the frequency crossover, and C governs the decay at high frequencies. The PSD profiles represent averages of four AFM measurements per condition, and the four ABC fits yielded *R*^2^ values of 0.9947–0.9966 when evaluated on a log(PSD) scale. To quantify changes after aging, the absolute differences between the fresh and aged fitted profiles were integrated over their common fitting interval of 0.0015–0.035 nm^−1^ and normalized by the integral of the fresh fitted profile. This comparison yielded integrated absolute deviations of 13.5% for the NFA-rich PM6 active layer and 4.1% for the g-HTL, supporting limited changes in the fitted g-HTL surface PSD profile within the analyzed interval. As discussed in our recent report, the reduced polymer content in NFA-rich blends weakens the kinetic constraints imposed by the polymer during film formation and allows NFA molecules to form ordered and crystalline morphologies at an early stage [25]. This behavior is consistent with an NFA-rich morphology closer to a quasi-equilibrium state and less susceptible to thermally driven structural reorganization. Such morphological stability is expected to help maintain the donor–acceptor interfacial structure and the interconnected charge-transport pathways established in the fresh film during prolonged thermal operation [49]. For the g-HTL, the small integrated deviation between the fresh and aged fitted PSD profiles, together with the limited changes in *R*_q_, provides complementary evidence supporting the stability of its surface morphology under prolonged thermal stress.

The combined photovoltaic and morphological results provide insights into the origin of the simultaneous enhancement in efficiency and thermal stability achieved with the g-HTL. In OPVs employing a thermally evaporated MoO_3_ HTL, the active layer/HTL interface is often vulnerable to thermal degradation because the MoO_3_ species may interact with or penetrate the upper region of the organic active layer during deposition, and their distribution can evolve further during subsequent thermal aging. Such interfacial evolution modifies the local electronic states, increases trap-assisted recombination, and contributes to burn-in degradation in MoO_3_-based devices [7,13,22]. By contrast, the formation of a g-HTL prevents direct thermal evaporation of an oxide onto the active layer while establishing favorable hole-transport and extraction characteristics, as evidenced by the higher effective hole mobility, lower initial trap density, enhanced *J*_ph_/*J*_sat_ at low *V*_eff_, and reduced *V*_OC_ dependence on light intensity. These measurements on fresh devices explain the improvement in initial photovoltaic performance. To assess resistance to thermally induced electronic degradation, *N*_t_ was additionally measured before and after 60 h of thermal aging at 85 °C (Figure S6). Averaged over four samples per condition, *N*_t_ increased from 2.8 (± 0.17) × 10^16^ to 3.0 (± 0.14) × 10^16^ cm^−3^ for the g-HTL devices. For the MoO_3_-based devices, it increased from 3.8 (± 0.31) × 10^16^ to 5.6 (± 0.25) × 10^16^ cm^−3^. The smaller mean increase in the g-HTL devices supports greater resistance to trap accumulation under thermal stress. AFM and PSD analyses further showed that both the NFA-rich active layer and the graded region formed from printed PM6 underwent limited changes in surface morphology after 15 days of thermal aging at 85 °C. Together, these electronic and morphological changes observed during aging provide complementary evidence supporting the enhanced thermal stability of the g-HTL devices. Accordingly, these results support the interpretation that the superior thermal durability of the g-HTL OPVs arises from the combined effects of circumventing a thermally vulnerable MoO_3_/BHJ interface and preserving the surface morphologies of both the active layer and polymeric hole-transport region. These complementary factors suppress the initial burn-in loss and slow the subsequent performance degradation, enabling the g-HTL devices to maintain high photovoltaic performance over an extended aging period. Together with its solution-based, vacuum-free, and potentially scalable fabrication process, this graded interfacial architecture provides a promising strategy for fabricating durable OPVs and other organic optoelectronic devices requiring stable hole-selective contacts.

## 4. Conclusions

We demonstrated that a diffusion-driven graded polymer film is an effective hole-selective contact material for thermally durable OPVs. By combining water-transfer printing with limited-depth PMA surface doping, the printed PM6 layer was converted into a vertically graded p^+^–p–i structure while retaining a laterally correlated surface morphology. The resulting interfacial architecture facilitated hole transport and carrier extraction while reducing trap-related recombination at the hole-collecting side. The smaller aging-induced increase in trap density and limited changes in the surface morphologies of both the NFA-rich active layer and printed PM6-based transport region provide complementary electronic and morphological evidence supporting the enhanced stability of the g-HTL devices. In parallel, replacing the thermally evaporated MoO_3_ avoids the formation of a thermally vulnerable oxide/organic interface that could otherwise undergo interfacial degradation and contribute to burn-in loss. Therefore, the enhanced thermal durability of the g-HTL OPVs is attributed to the combined effects of reduced trap accumulation under thermal stress, preservation of surface morphology, and avoidance of a thermally vulnerable MoO_3_/BHJ interface. These findings indicate that controlled molecular diffusion is an effective strategy for engineering graded polymeric hole-selective interfaces without relying on vacuum-deposited metal oxides. More broadly, the results demonstrate that integrating a favorable depth-dependent electronic structure with a thermally stable morphology and print-compatible processing offers a practical design principle for sustaining efficient charge extraction during prolonged operation. This interfacial concept offers a promising route toward efficient and durable OPVs and can be extended to other optoelectronic devices that require stable charge-selective contacts.

## Data Availability

Data is contained within the article or Supplementary Materials.

## Supplementary Materials

The following supporting information can be downloaded at: https://www.mdpi.com/article/10.3390/polym18212636/s1, Figure S1: Work functions of pristine and PMA-treated printed PM6 films; Figure S2: Depth-resolved Mo 3d and Mo 3p/N 1s spectra of the printed PM6 film without PMA treatment at selected etching times; Figure S3: Statistical histograms of photovoltaic parameters for 18 independently fabricated g-HTL OPVs; Figure S4: AFM topography and phase images of the NFA-rich PM6:L8-BO active layer and the g-HTL before and after thermal aging at 85 °C for 15 days; Figure S5: Topographic and phase *R*_q_ values of the NFA-rich PM6:L8-BO active layer and g-HTL before and after thermal aging at 85 °C for 15 days; Figure S6: *N*_t_ of MoO_3_ and g-HTL devices before and after 60 h of thermal aging at 85 °C.
