# Supplementary material for: Molecular Diffusion-Driven Graded Polymer Hole-Transport Layers Enabling Efficient and Durable Organic Photovoltaics
Source: Polymers (Basel). 2026 Oct 24;18(21):2636. doi: 10.3390/polym18212636 (PMC13765544; doi:10.3390/polym18212636)
Supplement: Supplementary file 1 [file polymers-18-02636-s001.zip › polymers-4626675-supplementary.pdf]

polymers

MDPI

Supplementary Material

Molecular Diffusion-Driven Graded Polymer Hole-Transport Layers Enabling Efficient and Durable Organic Photovoltaics

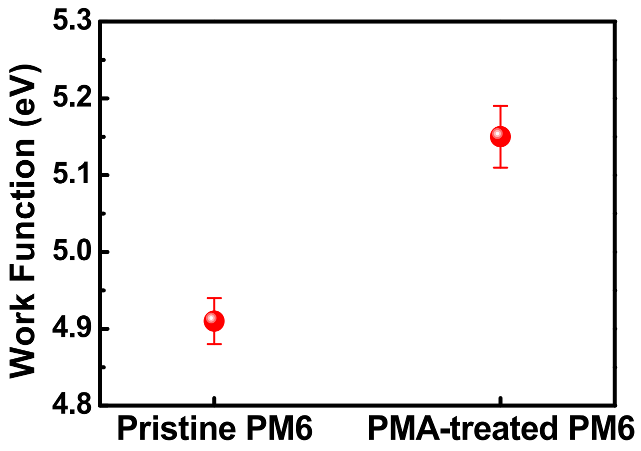

Figure S1. Work functions of pristine and PMA-treated printed PM6 films. Symbols represent mean values, and error bars indicate statistical deviations from four independent measurements.

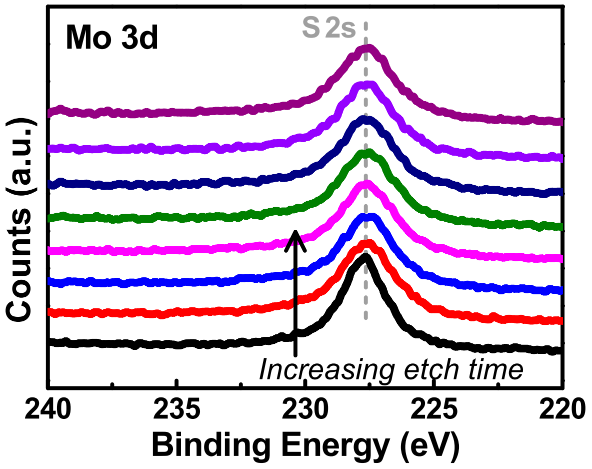

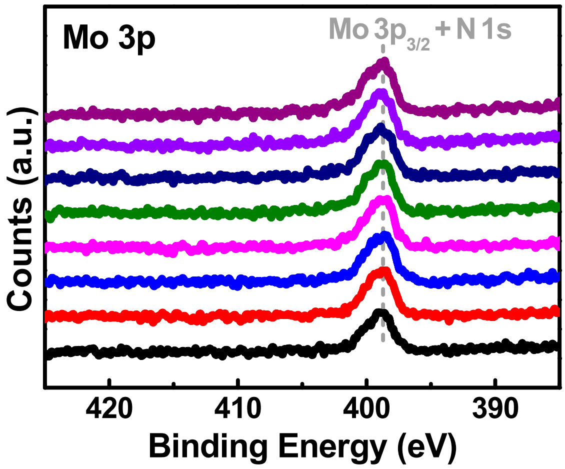

Figure S2. Depth-resolved Mo 3d and Mo 3p/N 1s spectra of the printed PM6 film without PMA treatment at selected etching times. The spectra are vertically offset for clarity, with increasing etching time corresponding to increasing analysis depth.

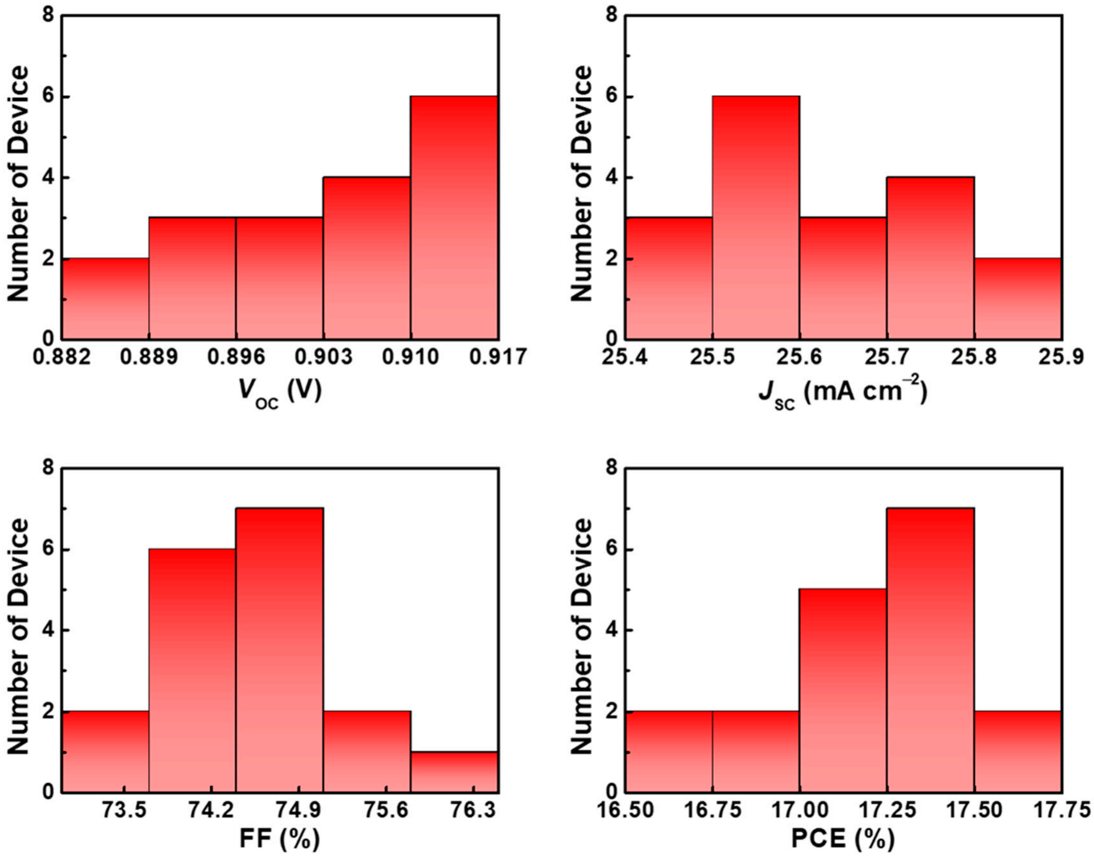

Figure S3. Statistical histograms of photovoltaic parameters for 18 independently fabricated g-HTL OPVs. Each parameter is divided into five equal-width bins, with the bin width selected separately for each parameter. The vertical axes indicate the number of devices in each bin.

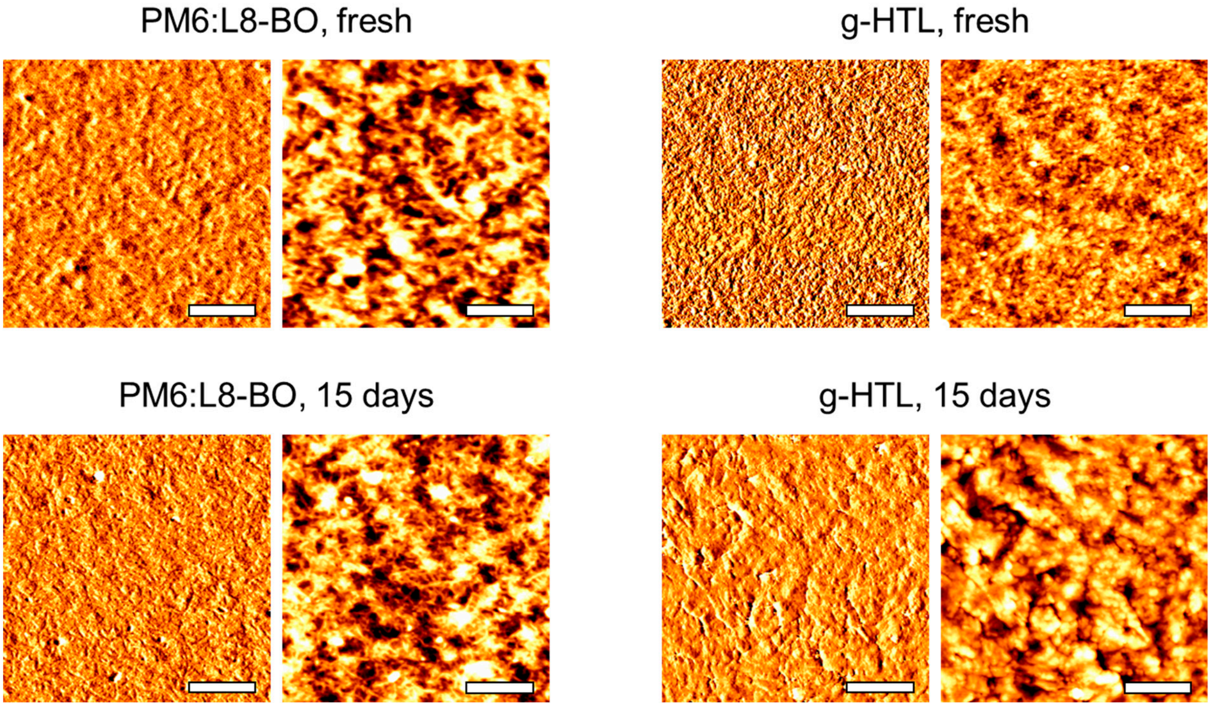

Figure S4. AFM topography and phase images of the NFA-rich PM6:L8-BO active layer and the g-HTL before and after thermal aging at 85 °C for 15 days. Scale bars: 500 nm.

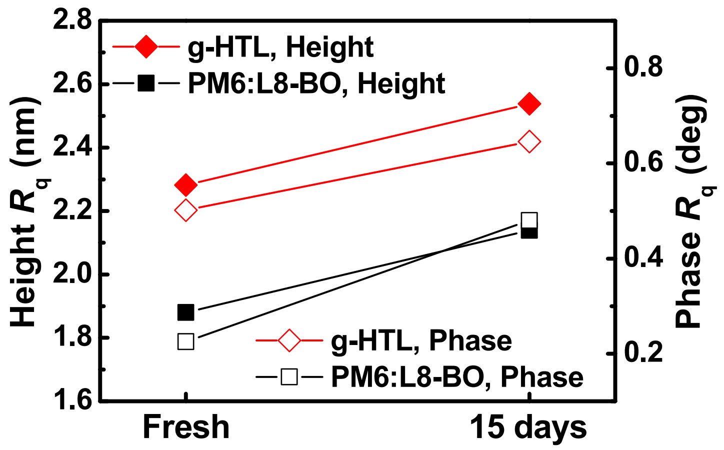

Figure S5. Topographic and phase R_q values of the NFA-rich PM6:L8-BO active layer and g-HTL before and after thermal aging at 85 °C for 15 days.

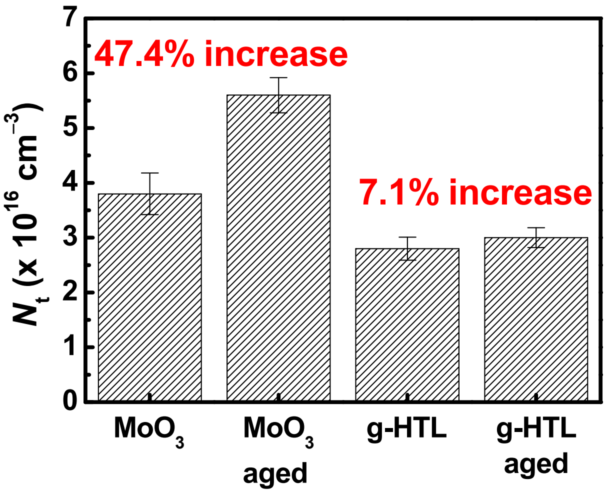

Figure S6. N_t of MoO_3 and g-HTL devices before and after 60 h of thermal aging at 85 °C. Bars represent mean values from four samples per condition, and error bars indicate standard deviations. The percentages denote the relative increases in mean N_t after aging compared with the corresponding fresh values.
